# Contralesional Brain–Computer Interface Control of a Powered Exoskeleton for Motor Recovery in Chronic Stroke Survivors

**DOI:** 10.1161/STROKEAHA.116.016304

**Published:** 2017-06-26

**Authors:** David T. Bundy, Lauren Souders, Kelly Baranyai, Laura Leonard, Gerwin Schalk, Robert Coker, Daniel W. Moran, Thy Huskey, Eric C. Leuthardt

**Affiliations:** From the Department of Rehabilitation Medicine, University of Kansas Medical Center, Kansas City (D.T.B.); Departments of Biomedical Engineering (D.T.B., R.C., D.W.M., E.C.L.), Neurology (L.S., K.B., L.L., T.H.), Neurological Surgery (E.C.L.), Mechanical Engineering and Material Sciences (E.C.L.), and Neuroscience (E.C.L.), Washington University, St. Louis, MO; and National Center for Adaptive Neurotechnologies, Wadsworth Center, NYS Department of Health, Albany, NY (G.S.).

**Keywords:** arm, brain-computer interface, hand, rehabilitation, stroke

## Abstract

**Background and Purpose—:**

There are few effective therapies to achieve functional recovery from motor-related disabilities affecting the upper limb after stroke. This feasibility study tested whether a powered exoskeleton driven by a brain–computer interface (BCI), using neural activity from the unaffected cortical hemisphere, could affect motor recovery in chronic hemiparetic stroke survivors. This novel system was designed and configured for a home-based setting to test the feasibility of BCI-driven neurorehabilitation in outpatient environments.

**Methods—:**

Ten chronic hemiparetic stroke survivors with moderate-to-severe upper-limb motor impairment (mean Action Research Arm Test=13.4) used a powered exoskeleton that opened and closed the affected hand using spectral power from electroencephalographic signals from the unaffected hemisphere associated with imagined hand movements of the paretic limb. Patients used the system at home for 12 weeks. Motor function was evaluated before, during, and after the treatment.

**Results—:**

Across patients, our BCI-driven approach resulted in a statistically significant average increase of 6.2 points in the Action Research Arm Test. This behavioral improvement significantly correlated with improvements in BCI control. Secondary outcomes of grasp strength, Motricity Index, and the Canadian Occupational Performance Measure also significantly improved.

**Conclusions—:**

The findings demonstrate the therapeutic potential of a BCI-driven neurorehabilitation approach using the unaffected hemisphere in this uncontrolled sample of chronic stroke survivors. They also demonstrate that BCI-driven neurorehabilitation can be effectively delivered in the home environment, thus increasing the probability of future clinical translation.

**Clinical Trial Registration—:**

URL: http://www.clinicaltrials.gov. Unique identifier: NCT02552368.

## Abstract 2

Supplemental Digital Content is available in the text.

A significant challenge in the treatment of stroke survivors is the rehabilitation of chronic motor disabilities. Although behavioral therapies such as constraint-induced movement therapy^1^ or robot-aided sensorimotor stimulation^2^ can improve upper-limb motor function, they require some level of peripheral motor function to engage with the therapy. This residual function is variable across patients and absent in the setting of complete hemiplegia. An alternative to behavioral therapies is to engage with the patient’s central nervous system directly. Specifically, a brain–computer interface (BCI) system can measure movement-related signals from the central nervous system and provide meaningful feedback to the central nervous system to direct plasticity.

BCIs have recently emerged as novel and potentially powerful tools to restore function in chronic stroke survivors.^3^ Early results present promising demonstrations that BCI-controlled orthoses or functional electric stimulators can lead to improvements in motor function in chronic stroke survivors.^3–8^ These stroke-specific BCI systems for rehabilitation have focused on signals stemming from perilesional cortex, contralateral to the affected hand for BCI control. Because the ability to modulate perilesional cortical activity decreases with increasing cortical damage,^9^ it may be particularly important for neurorehabilitation systems to focus on the ipsilateral, contralesional cortex in those patients who are most severely affected.

Although movement-related neural activity occurs in the ipsilateral and the contralateral cortices,^10,11^ the role of the unaffected hemisphere in stroke recovery is uncertain. Specifically, decreases in contralesional activity are associated with optimal recovery in some studies.^12,13^ Other studies show that increases in contralesional activity may be related to motor recovery,^14,15^ particularly in patients with incomplete recovery.^16^ As motor recovery is inversely correlated with the extent of corticospinal tract transection,^17^ we hypothesized that using contralesional hemisphere activity to drive a BCI-controlled exoskeleton may lead to functional improvements. Previously, we demonstrated that chronic stroke survivors can control BCIs using electroencephalographic (EEG) signals from the contralesional hemisphere associated with the intention to move the affected limb.^18^ However, it was uncertain whether emphasizing the relationship between activation of ipsilateral cortex and resultant sensory feedback would be beneficial.

This feasibility study tested an EEG-BCI system that used signals related to affected hand motor imagery, recorded from the unaffected hemisphere, to control the affected hand via a powered exoskeleton. This study is the first to specifically focus on the unaffected hemisphere with a BCI rehabilitation system and the first to provide BCI-driven therapy in the patients’ homes. This setting is important because it increases the likelihood that this approach can be scaled more widely across the stroke-affected population.

## Methods

To determine whether a BCI-controlled exoskeleton using EEG signals from the unaffected hemisphere can lead to functional rehabilitation, we created a novel home-based system called the IpsiHand. We then examined whether a 12-week training period led to functional improvements in chronic, hemiparetic stroke survivors.

### Patient Characteristics

Ten chronic hemiparetic stroke survivors with moderate-to-severe upper-limb hemiparesis, enrolled at least 6 months after first-time hemispheric stroke, completed the study. Because motor recovery plateaus after 3 months,^19^ the study was designed as a self-controlled study comparing motor function before and after the intervention to establish the feasibility of the BCI-driven therapy studied. The Table contains patient demographics and baseline motor function. The online-only Data Supplement contains detailed inclusion and exclusion criteria. Moderate-to-severely impaired patients were specifically targeted because they are less likely to recover through other methods and therefore require an alternative rehabilitation strategy, such as a BCI. The Washington University School of Medicine Institutional Review Board approved the study protocol, and all patients provided written informed consent.

**Table.**
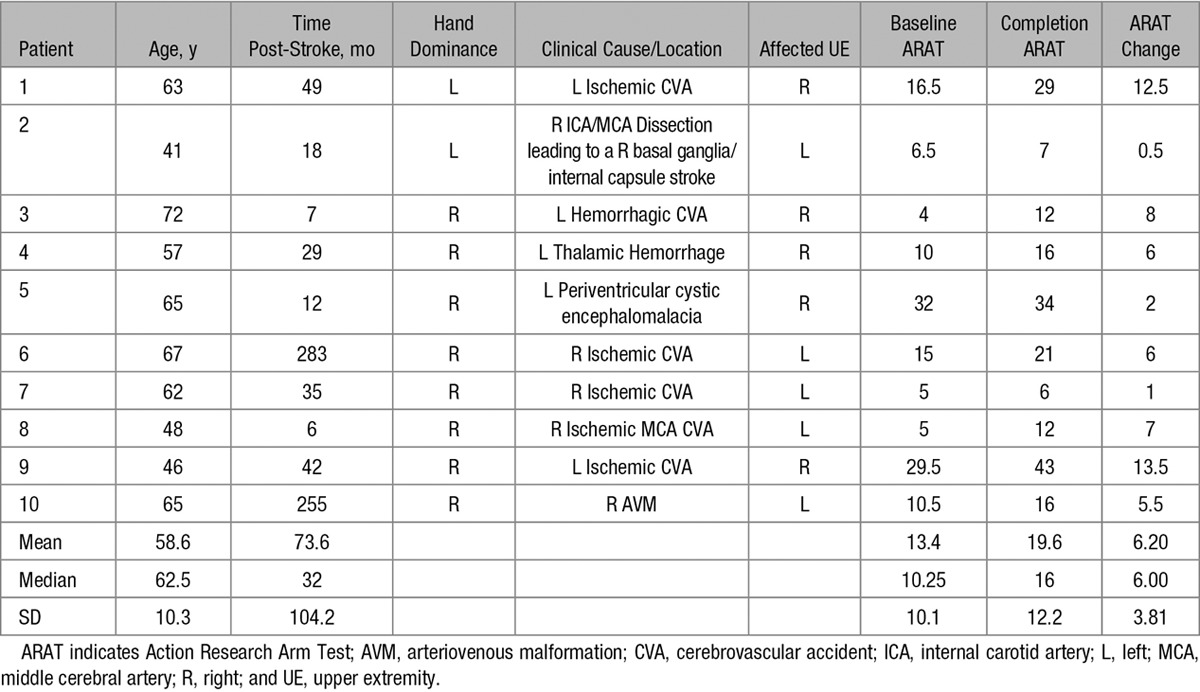
Patient Characteristics and ARAT Scores

### BCI System Design

**Figure 1.**
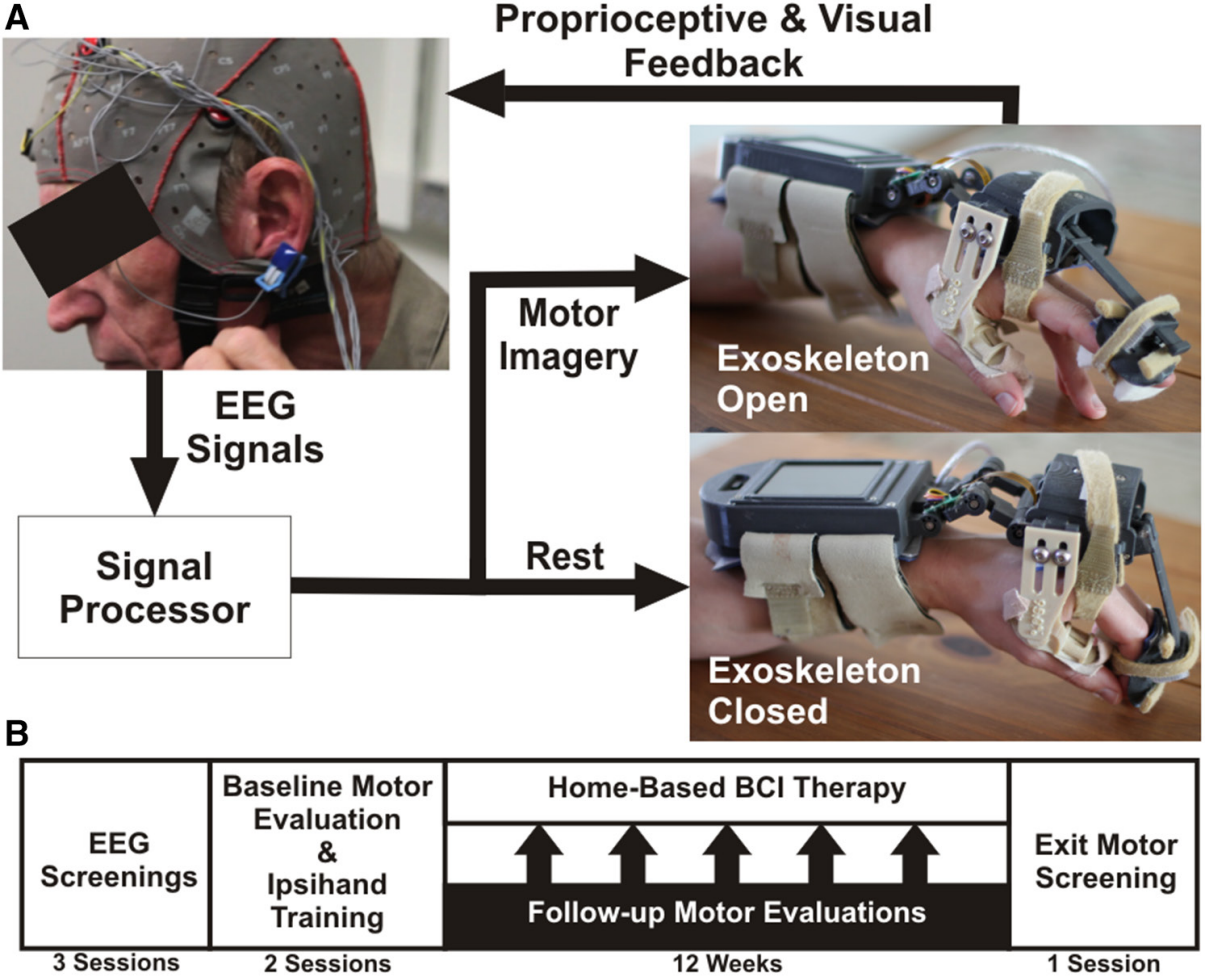
Study methodology. **A**, The exoskeleton used attached to a patient’s affected hand via straps on the forearm, palm of the hand, and intermediate phalanges of the index and middle finger, whereas the thumb was held stationary. The exoskeleton was controlled by a microprocessor in the forearm assembly that processed electroencephalographic (EEG) signals. A linear actuator drove hand movements in a 3-finger pinch grip based on the decoded EEG. **B**, The study tested whether training with the brain–computer interface (BCI)–controlled exoskeleton would lead to functional improvements. Patients that met the inclusion criteria completed 3 EEG screenings. Patients with consistent movement-related EEG activations then completed baseline motor evaluations and BCI system training. Finally, patients completed a 12-wk home-based BCI protocol with follow-up motor evaluations at 2-wk intervals.

The BCI system (Figure 1A) combined a novel powered exoskeleton with a commercial EEG amplifier and active electrodes. The exoskeleton opened and closed the patient’s hand in a 3-finger pinch grip (1 degree of freedom). A detailed description of the system is contained in the Methods in the online-only Data Supplement. Consistent with our previous work,^18^ the system used spectral power changes to control hand position. Because stroke patients typically have difficulty extending their extremities, BCI control associated motor imagery with opening the affected hand. Each trial began with the hand fully closed, and spectral power at the control feature was used to update the hand position, providing visual and proprioceptive feedback. During rest trials, patients were instructed to try to keep the exoskeleton closed by imagining that they were resting. During movement trials, patients were instructed to try to open their hand via motor imagery.

### EEG Screening

After meeting the inclusion criteria, patients underwent an EEG screening protocol to ensure that a consistent control signal was present for device control. Each patient completed 3 separate screenings to assess the stability of potential BCI control signals. EEG electrodes were applied by a trained biomedical engineer, and EEG signals were collected while patients performed a visually cued motor screening task consisting of trials of (1) rest, (2) unaffected hand movements, (3) affected hand motor imagery, and (4) bilateral motor imagery. Spectral power, or the power in the EEG signal as a function of frequency, was calculated using an autoregressive spectral estimation method. The coefficient of determination (*r*^2^), the percent of variance in spectral power that was accounted for by the difference between affected hand motor imagery and rest trials, was calculated for each channel and frequency. After completing 3 EEG screenings, the EEG data were examined for the presence of consistent spectral power changes during affected hand motor imagery. BCI control features were required to be associated with imagined movements of the affected hand and located in unaffected hemisphere motor regions. These sessions were not designed to achieve BCI mastery but to identify patients with consistent cortical activations (ie, μ [8–12 Hz] or β (12–30 Hz) power decreases) in at least 2 of 3 sessions. The feature in the unaffected hemisphere with the strongest *r*^2^ value was chosen as the patient-specific BCI control feature. Patients without consistent spectral power changes were unable to continue in the study.

### Outcome Measures

The primary outcome measure was the Action Research Arm Test (ARAT).^20^ Secondary outcome measures included: (1) the Canadian Occupational Performance Measure,^21^ (2) the Motricity Index, (3) the modified Ashworth Scale at the elbow joint, (4) grip strength, (5) pinch strength, and (6) the active range of motion (AROM) at the metacarpophalangeal joint of digits 2 to 5. As this study was the first to use a BCI system for stroke rehabilitation in the home setting, we measured the BCI control quality by comparing the topographies of spectral power changes in the laboratory and home-based sessions. We assessed compliance by recording the total number of days and time that each patient used the system.

### Study Protocol

The study timeline is shown in Figure 1B. After completing the EEG screenings, patients completed 2 pretherapy motor evaluations in which all primary and secondary outcome measures were measured by an occupational therapist. On these days, the exoskeleton was also fit to the patient’s hand. In addition, patients and their caregivers were trained to use the system. This included (1) donning the exoskeleton and EEG cap, (2) examining the EEG readouts to verify that physiological signals were collected, (3) software operation, and (4) system maintenance. After the baseline motor evaluations and training, each patient was sent home with a BCI system to complete 12 weeks of training. Patients were instructed to use the BCI system on a minimum of 5 days per week. Patients completed 1 to 12 10-minute runs of the BCI task per day depending on their stamina and time constraints. At 2-week intervals, patients came to the laboratory for follow-up motor evaluations consisting of the ARAT and Canadian Occupational Performance Measure. At these follow-up sessions and as needed, an occupational therapist or a biomedical engineer communicated with the patients to ensure compliance with the study, answer questions about the device, fix any malfunctions, and discuss EEG signal quality, which was assessed regularly by a biomedical engineer. After 12 weeks, patients were again tested on all primary and secondary outcome measures. Different occupational therapists collected baseline and completion outcome measures, and all occupational therapists were blinded to observed EEG changes.

### Analysis of Outcome Measures

A paired-sample *t* test was used to evaluate the statistical significance of ARAT changes and continuous secondary outcome measures (grip strength, pinch strength, and AROM). Signed-rank tests were used for all other outcome measures because their measurement scales were ordinal. Because the exoskeleton drove extension of the second and third digits, AROM values for the second and third digits and fourth and fifth digits were averaged separately. Changes were examined for both the overall and subcomponents of the ARAT and Motricity Index.

### Neurophysiological Correlates

To examine potential mechanisms of action, we calculated the correlation between the change in ARAT and changes in BCI control accuracy, total usage time, and EEG modulation changes. To quantify BCI performance, we calculated the average hand position in the second half of each trial. The BCI accuracy for each run of the BCI task was calculated by taking the difference in this average position between movement and rest trials. EEG modulation was determined by calculating the coefficient of determination (*r*^2^ value) quantifying the difference in EEG spectral power between motor imagery and rest trials. The change in BCI accuracy and EEG modulation was defined as the slope of a robust multilinear regression representing the change per run of the BCI task. The relationship between the ARAT change and change in both BCI control accuracy and EEG modulation was measured with Spearman *r*. To control for the location and frequency used for BCI control, we performed 3 control analyses: (1) change in EEG modulation at the same frequency but at the location contralateral to the control site (ipsilesional motor cortex), (2) change in EEG modulation at the same frequency used for control but at a nonmotor electrode site (F3), and (3) change in EEG modulation at the location used for BCI control (contralesional motor cortex) but at a different frequency (50 Hz). Because patients performed the BCI task at home, poor-quality EEG activity was observed on some days. Thus, we included only those runs in which BCI control signals significantly (*P*<0.01) differed between movement and rest trials.

## Results

Ten patients completed the study. Patient characteristics are summarized in the Table, and the online-only Data Supplement contains a detailed description of patient recruitment. In short, of the 22 patients who completed EEG screenings, 18 (81%) were suitable for further BCI therapy, 13 (59%) began the therapy, and 10 (45%) completed the study. The drop off was because of a variety of causes, including unrelated medical diagnoses, inability to comply with the time commitment, and poor orthosis fit.

### BCI Control

**Figure 2.**
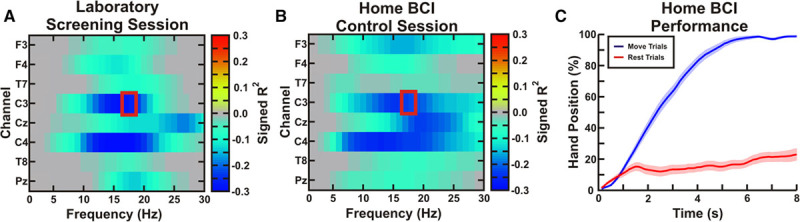
Exemplar electroencephalographic (EEG) activity and brain–computer interface (BCI) control. **A**, During an exemplar laboratory-based screening session, the patient (patient 10, left affected) demonstrated significant decreases in μ- and β-band spectral power bilaterally. The color scale shows signed *r*^*2*^ values indicating increases (positive values) and decreases (negative values) in spectral power during motor imagery. A BCI control feature (red box) ipsilateral to the affected hand was chosen (contact C3). **B**, During a home-based BCI control session, a similar spatiospectral pattern of movement-related EEG activity was observed. **C**, The mean (±SE) of the hand position in movement and rest trials shows that the patient achieved a high level of BCI control (0% fully closed, 100% fully open).

After initial training, patients and their caregivers were able to apply EEG electrodes in the home setting to record physiological EEG signals. Figure 2 shows exemplary movement-related EEG activity observed in the laboratory and while at home. The patient demonstrated bilateral μ- and β-band power decreases in both settings. Furthermore, the patient had very similar spatial and spectral patterns of movement-related EEG activity during both sessions. The significant decrease in power during motor imagery in the BCI control task led to a high level of accuracy with discriminable patterns of exoskeleton movement during rest and motor imagery.

Because our hypothesis focused on the contralesional hemisphere, the features used to drive the BCI system were from electrodes over the contralesional motor cortex. Movement-related EEG activations were also observed from the ipsilesional hemisphere in 8 of the 10 patients. Although the frequency used for BCI control varied across patients, all BCI control features were μ- and β-band power suppressions, also referred to as event-related desynchronization.^22^ Patients used the device on 37 to 72 days. Patients performed 74 to 465 10-minute runs of the BCI task for a total of 740 to 4650 minutes of online BCI control in addition to the daily screening task. Details of the patient-specific BCI control are included in the online-only Data Supplement.

### Functional Outcomes

**Figure 3.**
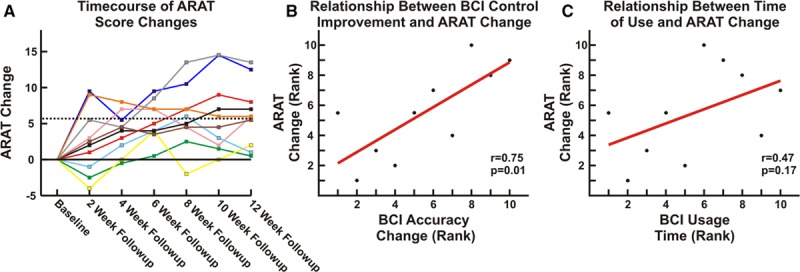
Improvement in motor function. **A**, Each line shows the change in Action Research Arm Test (ARAT) during the study. At completion, 6 of 10 patients had ARAT increases surpassing the minimal clinically important difference (MCID; 5.7 points). **B**, ARAT increases were related to the rate of change in brain–computer interface (BCI) accuracy (Spearman *r*=0.75, *P*=0.013). **C**, ARAT increases were not related to the time of device use (Spearman *r*=0.47, *P*=0.17).

**Figure 4.**
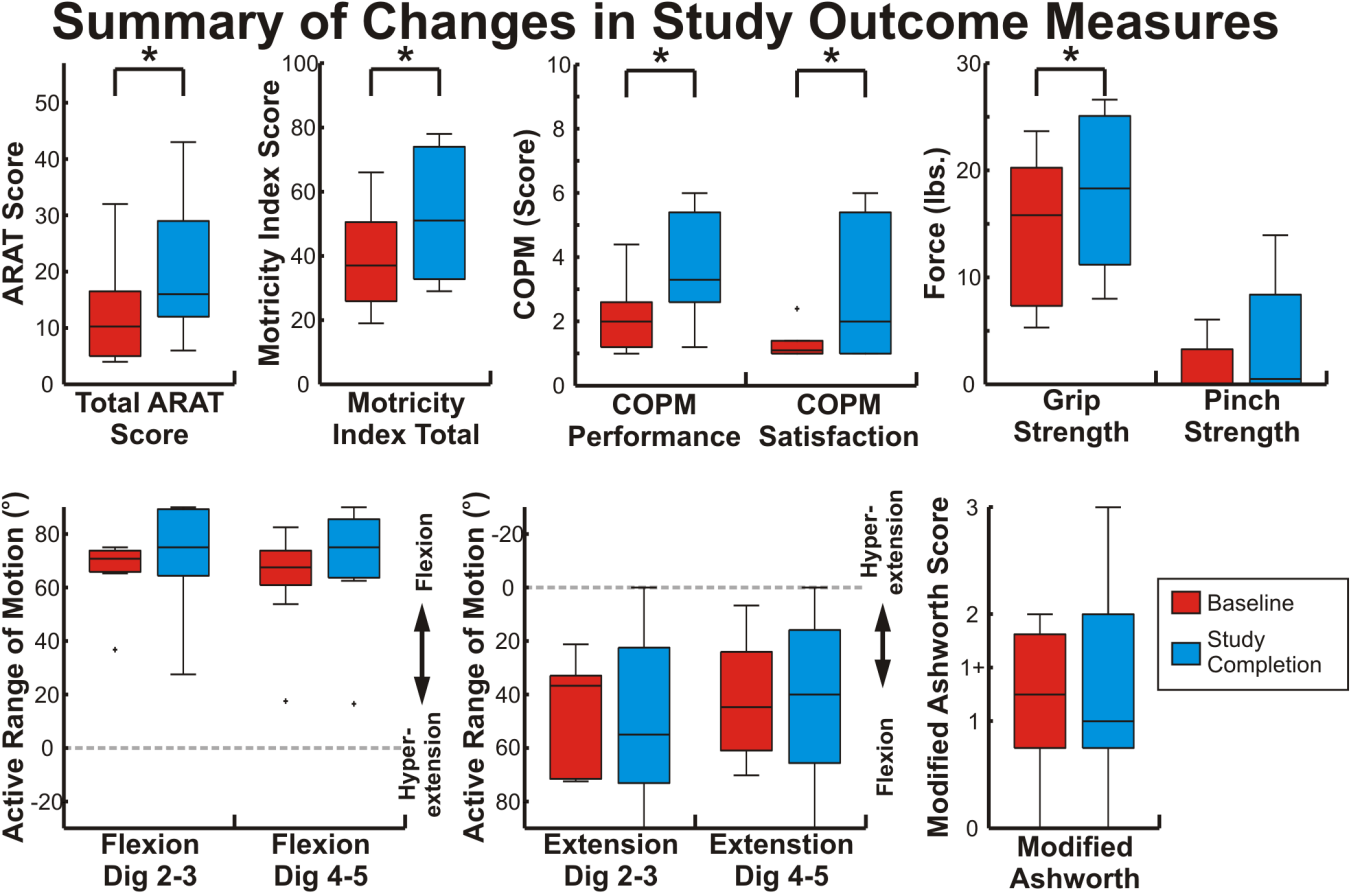
Summary of outcome measures. Each box shows the distribution of each outcome measurement at baseline and study completion. Boxes show the 25th percentile, median, and 75th percentile; bars indicate the range of values; and outliers >2.7 SDs from the mean are marked with a +. Measures with statistically significant (*P*<0.05) changes are indicated with an *. ARAT indicates Action Research Arm Test; and COPM, Canadian Occupational Performance Measure.

The 2 baseline motor assessments were averaged to determine each patient’s baseline motor function. ARAT changes throughout the study protocol are shown in Figure 3A. Patients had a statistically significant mean ARAT increase of 6.2 points. Importantly, 5.7 points has been estimated to represent the minimal clinically important difference in chronic stroke survivors.^23^ Specifically, 6 of the 10 patients had ARAT improvements above this level. In addition to this per-protocol analysis, a significant increase in ARAT score was also found using an intention-to-treat analysis as described in the online-only Data Supplement. Grasp strength, Motricity Index, the grip and grasp ARAT subscores, and Canadian Occupational Performance Measure performance and satisfaction ratings were also significantly increased after therapy, whereas pinch strength, AROM, and the pinch and gross ARAT subscores were not changed. Figure 4 and Table II in the online-only Data Supplement summarize changes across outcomes. Other than minor fatigue, no negative effects were observed.

### Neurophysiological Correlates

Across patients, there was a significant correlation between the change in ARAT score and the change in BCI accuracy (defined as the difference between the hand position in the movement and rest trials) per BCI task run (Figure 3B; Spearman *r*=0.75, *P*=0.013). There was not a significant relationship between the change in ARAT score and the total device usage time (Figure 3C; Spearman *r*=0.47, *P*=0.17).

**Figure 5.**
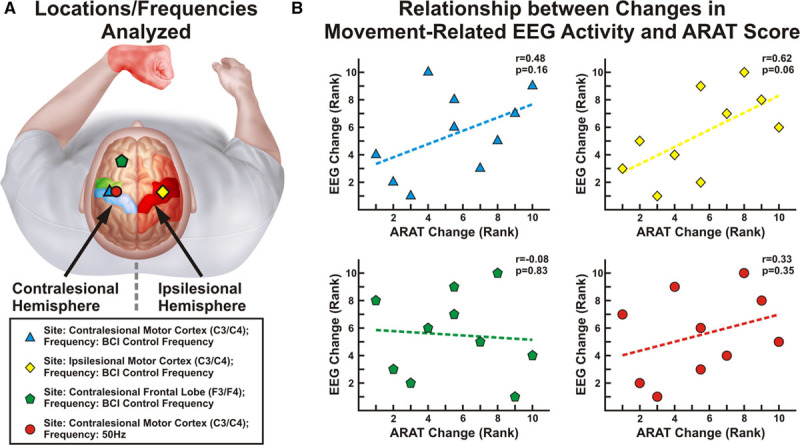
Relationship between changes in electroencephalographic (EEG) activity and Action Research Arm Test (ARAT) improvements. Ranked changes in motor function (ARAT) and changes in EEG activations (*r*^*2*^ value) per brain–computer interface (BCI) run are shown. **A**, Analyses were performed using EEG activity at the site and frequency used for BCI control, at the frequency used for BCI control but an electrode in the contralateral hemisphere, at the frequency used for BCI control but an electrode in the frontal lobe (F3; serving as a spatial control), and at the site used for BCI control but at 50 Hz (serving as a spectral control). **B**, There was a positive relationship that trended toward significance at both the BCI control feature (**top left**) and in the contralateral motor cortex (**top right**) but not at a location outside the motor cortex (**bottom left**) or a task-irrelevant frequency (**bottom right**).

Finally, we sought to determine whether there was a relationship between ARAT and EEG changes (Figure 5). There was a trend toward a positive relationship between ARAT score changes and the change in the EEG modulation per run of the BCI task at the location and frequency used for BCI control and in a site in the contralateral motor cortex (BCI control feature: Spearman *r*=0.48, *P*=0.16, contralateral motor cortex: Spearman *r*=0.62, *P*=0.06).

## Discussion

This study provides evidence for the potential role of the unaffected hemisphere in rehabilitation via a BCI-controlled exoskeleton. Specifically, patients had an average ARAT improvement surpassing the minimal clinically important difference.^23^ In addition, improvements were observed in some, but not all, objective secondary measures of function. Although pinch strength, AROM, and the ARAT pinch subcomponent did not change, these measures are less sensitive in more severely impaired patients and were likely affected by a qualitative increase in spasticity observed, particularly in patients who had received botox 90 to 120 days before study onset. Furthermore, the grasp and grip ARAT subcomponents and grip strength, which all involve distal hand function, significantly improved. It is uncertain whether observed improvements in general distal hand function that did not localize to pinch were because of the poor spatial specificity of EEG or the sensitivity of pinch-specific subcomponents. Finally, we also observed statistically significant increases in a self-scored subjective measure of each patient’s use of their affected arm in functional tasks (Canadian Occupational Performance Measure). These findings build on previous evidence that BCI-controlled rehabilitation systems can facilitate motor recovery.^4–8^ There are several features that distinguish this work from previous studies. First, this study was the first to focus exclusively on using the unaffected hemisphere in a BCI rehabilitation system. Second, the BCI drove the velocity of the exoskeleton, providing a closer temporal pairing between brain activity and proprioceptive feedback than previous systems.^4,6^

The choice of a BCI control signal for poststroke motor rehabilitation requires careful consideration, particularly given the conflicting evidence on the unaffected hemisphere after stroke.^12–15,24–28^ By pairing cortical activations with peripheral feedback, we hypothesized that we would induce plasticity in the remaining (ipsilateral) central nervous system pathways. As noted, there was a significant relationship between the change in ARAT scores and the rate of change in BCI control accuracy that could not be explained by the volume of device use. Further, there was a trend toward a significant relationship between the rate of change in EEG activity and ARAT score specific to the bilateral motor system, but not in the frontal lobe or at task-irrelevant frequencies. Therefore, although what can be asserted from a mechanistic standpoint is somewhat limited, the results indicate that the choice of a BCI control feature in the unaffected hemisphere may have played an important role in the benefits of the intervention.

There are many potential explanations that could account for the functional improvements observed. Specifically, although postrecovery increases in activity have been found in both the affected and unaffected hemispheres,^16,24,26,29^ the reorganization of interhemispheric connectivity between the contralesional and ipsilesional motor cortices may also play a role in functional recovery.^17,28^ Further studies designed to better define the mechanism of action will be beneficial to better understand the characteristics of patients who will benefit optimally from BCIs controlled from the unaffected hemisphere. Because the integrity of the ipsilesional corticospinal tract is strongly correlated with motor recovery,^17^ we would hypothesize that the corticospinal tract integrity is essential in determining what role the contralesional hemisphere will play in recovery. Specifically, in patients with the greatest corticospinal tract damage, we would expect recovery to require an alternative pathway, such as fibers descending ipsilateral to the contralesional motor cortex.

This study was also unique in that the system was used in the home setting without daily oversight. Traditional BCI systems for rehabilitation have been used in a laboratory setting with trained experts operating them.^4–8^ The ability to provide therapy in a patient’s home without constant supervision would likely reduce the cost of therapy, increase the time of therapy, and give patients flexibility in scheduling therapy. For this approach to achieve large-scale implementation, several practical aspects will need to be addressed, including building the system in a cost-effective fashion, optimizing the orthosis and EEG headset design for enhanced user experience and compliance, and integrating the hardware and software to enable seamless remote maintenance and minimize the need for EEG quality checks.

There are also several limitations to note. Because of the home-based setting, it was impossible to ensure that data were free from artifacts. Although the majority of patients had good-quality EEG recordings in the majority of sessions, a few patients met this standard in <50% of sessions. In addition, because the study sample is small in size and was restricted to those with enough motivation to complete the study protocol, the scope and generalizability of the results is uncertain. Also, pinch strength, all Motricity Index subcomponents, ARAT pinch and gross subcomponents, and AROM did not improve. Whether this was because of the poorer sensitivity of these subcomponents combined with the small sample size, the poor spatial resolution of the EEG signals used, or a limitation of the therapy is uncertain. Finally, the study was uncontrolled. Previous work has shown ARAT improvements can be achieved in chronic stroke patients after interventions such as constraint-induced movement therapy or standard physical therapy,^30^ but patients in these studies began with a much higher baseline ARAT score than the current cohort. Also of note, while shorter in duration (2 weeks), a randomized controlled trial of a BCI-controlled hand orthosis in patients with a similar baseline motor function showed no improvement in a control group receiving a sham therapy.^6^ Taken together, there remains an open question of whether more severely affected chronic stroke patients benefit from a BCI intervention exclusively versus prolonged physical therapy; a question that will ultimately be answered with a randomized clinical trial. However, this work provides important early evidence that training with a BCI-driven orthosis can be implemented in the home environment and is associated with a meaningful functional improvement.

## Conclusions

This feasibility study shows a statistically significant and clinically meaningful improvement in the motor function of chronic stroke survivors after using a home-based BCI-controlled exoskeleton. The use of control features in the contralesional hemisphere shows evidence of the potential relevance of the unaffected hemisphere for functional rehabilitation. Collectively, although this study represents an important step toward developing and translating BCI-driven rehabilitation protocols for chronic stroke survivors, the effectiveness of BCI-driven therapies must be proven in large randomized controlled trials before full acceptance.

## Sources of Funding

This study was funded by Neurolutions, Inc.

## Disclosures

Dr Bundy, Dr Schalk, R. Coker, Dr Moran, and Dr Leuthardt own stock in Neurolutions, Inc. Study data were reviewed by an unaffiliated neurologist before submission as part of a comprehensive conflict of interest management plan. The other authors report no conflicts.

## Supplementary Material
