# Supplementary material for: Contralesional Brain–Computer Interface Control of a Powered Exoskeleton for Motor Recovery in Chronic Stroke Survivors
Source: Stroke. 2017 Jun 26;48(7):1908–15. doi: 10.1161/STROKEAHA.116.016304 (PMC5482564; doi:10.1161/STROKEAHA.116.016304)
Supplement: Supplementary file 1 [file str-48-1908-s001.pdf]

RELEASE

I, the individual identified below (the "Individual"), hereby grant Neurolutions, Inc., a Delaware corporation (the "Company") and its assigns, successors in interest, employees, consultants, and those acting on behalf of the Company, the irrevocable unrestricted right and license to (a) take or record audio, pictures and video of me and (b) use, publicly display, distribute, perform, modify and create derivative works of, and use my name (or any fictional name), picture, portrait, likeness or photograph and audio and video in which I participate or appear, including videos produced in connection with my enrollment in the Company's clinical trial (collectively, the "Images"), in whole or in part and in any and all forms, media and manners now or hereafter known, including on the Company's website located at [illegible], any other website, social network or social or digital media that is now or heareafter owned by the Company, for educational, commercial, informational and any other purposes, including publicity and promotional purposes. I waive any right to inspect or approve any Images produced, any alterations thereto or any finished products incorporating my name and/or the Images.

I hereby release and hold harmless the Company from any damages or liability relating to or arising from any use of or modification or alteration to any of the Images. I waive any claim I may have based on any use of the Images or works derived therefrom, including but not limited to claims for invasion of privacy or libel or for financial or other compensation. I understand that the Company would not use the Images without first receiving a copy of this Release signed by me.

I represent and warrant that I have read this Release and am familiar with its contents. I further represent and warrant that I am 18 years or older and competent to sign this release. This Release shall survive the execution and delivery hereof and shall be binding on me, my legal representatives, heirs and assigns.

Name of Individual: Rick Arnold

Address of Individual: 1660 Turf Ln
FLORISSANT, MO. 63033-2344

Signature: [illegible]

Print Name: Rick Arnold
(please print)

Date: October 20, 2014
