# Supplementary material for: Contralesional Brain–Computer Interface Control of a Powered Exoskeleton for Motor Recovery in Chronic Stroke Survivors
Source: Stroke. 2017 Jun 26;48(7):1908–15. doi: 10.1161/STROKEAHA.116.016304 (PMC5482564; doi:10.1161/STROKEAHA.116.016304)
Supplement: Supplementary file 2 [file str-48-1908-s002.pdf]

SUPPLEMENTAL MATERIAL

Contralesional Brain-Computer Interface Control of a Powered Exoskeleton for Motor Recovery in Chronic Stroke Survivors

David T. Bundy PhD[1,2], Lauren Souders MOT[3], Kelly Baranyai MOT[3], Laura Leonard MOT[3], Gerwin Schalk PhD[5], Robert Coker MS[2], Daniel W. Moran PhD[2], Thy Huskey MD[3*], Eric C. Leuthardt MD[2,4*]

Affiliations:
[1]Department of Rehabilitation Medicine, University of Kansas Medical Center, Kansas City, KS
Departments of [2]Biomedical Engineering, [3]Neurology, [4]Neurological Surgery, Washington University, St. Louis, MO
[4]Wadsworth Center, NYS Department of Health, Albany, NY
[*]Contributed equally

Supplemental Methods

Inclusion/Exclusion Criteria

All patients were chronic, hemiparetic stroke survivors, defined as at least 6 months post first-time stroke. Motor recovery had plateaued and any standard rehabilitation therapy had been discontinued. Specific inclusion criteria consisted of moderate to severe impairment of the upper extremity, limited spasticity (Modified Ashworth score of 1+ or less), full passive range of motion of the affected elbow, wrist, and digits, and normal sensation of the affected upper extremity. As movement of the hand during the trial was completed by the mechanical orthosis, there was no baseline level of active motor control at any joint in the upper extremity required for participation. Full passive range of motion was required, however, in order to ensure that the orthosis could adequately drive hand movements. Exclusion criteria included severe visual impairment, cognitive impairment (8 or more on the Short Blessed Test), botox injections in the affected upper extremity for spasticity management in the prior 3 months, severe aphasia, ataxia, and unilateral neglect.

BCI System Design

The hand component of the exoskeleton was connected to a forearm assembly, which housed a controller board with a microprocessor, motor driver, and touchscreen display for user interface. The exoskeleton was attached to the patient's hand by straps around the forearm, palm of the hand, and the intermediate phalanges of the index and middle fingers. Because the system was designed for daily use by patients, a limited montage of electrode locations (F3, F4, T7, C3, Cz, C4, T8, Pz) was used. EEG signals were collected using commercially available g.LadyBird active electrodes system and a commercially available g.Mobilab+ EEG amplifier (g.Tec, Graz, Austria). Custom software was written to receive and buffer EEG signals, perform signal processing, and control the position of the exoskeleton. Additionally, the software provided instructions for the patients, received touchscreen inputs to start and stop sessions, and included a display of raw EEG signals to allow patients and researchers to verify that physiologic signals were being captured.

Screening Task

The screening task used to assess spectral power changes associated with motor imagery of the affected hand consisted of 8-second trials of: 1) rest, 2) unaffected hand movement, 3) affected hand motor imagery, and 4) bilateral motor imagery. Each run consisted of 12 trials of each condition and 4 runs were completed in each session for a total of 48 trials of each condition. Data from the screening session was analyzed offline by re-referencing EEG signals to the common average and using an autoregressive method for spectral power estimation known as the Maximum Entropy Method (MEM) to calculate spectral power in 1Hz bins from 1 - 50Hz using 500 msec sliding windows. Following the screening task, a single calibration run (30 affected hand motor imagery trials and 30 rest trials) was performed and served to validate the chosen BCI control feature.

BCI Control Sessions

During online BCI control sessions, EEG signals were re-referenced to the common average and spectral analysis was performed in 1 Hz bins on 500 msec windows of EEG data shifted by 125 msec per window using the MEM algorithm[1]. After each 500 msec

window was collected, the spectral power at the control feature was used to update the glove position as described by equation S1:

$$Y(t) = Y(t-1) + Gain\frac{(X(t) - \mu_{Rest} - Bias)}{\sigma_{Rest}} sign(\mu_{Move} - \mu_{Rest}) \qquad \text{(S1)}$$

where Y(t) is the current glove position constrained to the 0-100% range, Y(t-1) is the previous glove position, X(t) is the current value of the BCI control feature, μ_{rest} and μ_{move} are the means of the BCI control feature during the motor imagery and rest trials, σ_{rest} is the standard deviation of the BCI control feature during the rest trials, *Gain* is a gain term controlling the speed of the movement, and *Bias* is a bias term designed to improve the ability to discriminate rest periods.

Each run of the BCI control task consisted of 30 rest trials and 30 movement trials. Each trial was 8 seconds in duration. During rest trials, patients were instructed to try to keep their hand closed by imagining that they were resting and during movement trials, they were instructed to try to open the exoskeleton by performing motor imagery. During control, both visual and proprioceptive feedback of the current hand position was provided by the exoskeleton. Visually, position was displayed on the touchscreen attached to the patient's forearm in the form of a moving bar. Simultaneously, the actuator on the exoskeleton opened and closed the patient's hand based upon the spectral power from the BCI control feature. Patient usage data, including raw EEG signals and the corresponding hand position, were stored for later analysis.

Outcome Measures

The primary outcome measure was the Action Research Arm Test (ARAT). The ARAT is a 57-point test designed to assess specific changes in upper limb function with sub-components for grasp, grip, pinch, and gross motor movement[2, 3]. The ARAT is a standardized clinical test of arm and hand function used world-wide to quantify post stroke motor deficits in humans. This test has been validated across numerous studies[3-5] and found to be equally as sensitive to other commonly used tools such as the Fugl Meyer Assessment[6]. The Canadian Occupational Performance Measure (COPM) is an evidence-based outcome measure designed to capture a client's self-perception of performance in 5 patient-identified tasks over time[7]. At study onset, patients identified 5 functional activities that they wanted to perform more independently or with greater ease. COPM measurements consisted of a semi-structured interview in which patients self-rated their performance and satisfaction with each activity on an ordinal scale from 1 to 10. The Motricity Index provides an overall indication of a patient's limb impairment by grading pinch, shoulder abduction, and elbow flexion on an ordinal scale from 0-5 and reweighting the scale based upon the difficulty experienced by patients in progressing from one grade to the next[8]. Each joint is assigned a weighted score between 0 and 33 and the scores for the three joints are summed to produce a score between 0 and 100 for the affected upper limb. The modified Ashworth Scale measured spasticity on an ordinal scale from 0-4 with an additional intermediate level (1+) to make the scale more discrete[9]. Gross grasp grip strength and three-finger pinch grip strength were measured using dynamometers. Finally, active range of motion at the metacarpophalangeal joints of the affected hand was measured relative to full extension with a goniometer using standard protocols. Positive values for active range of

motion indicated a final position that was in flexion relative to full extension and negative values indicated a final position that was in hyperextension relative to full extension.

Study Protocol

Throughout the 12-week study period, patients were instructed to use the BCI system at a minimum level of 5 days per week. On each day, patients began by donning the EEG electrodes. Patients then completed a calibration task in which EEG signals were stored during 90 seconds of rest during which patients were instructed to remain still and 30 trials each of affected hand motor imagery and rest. After completing the calibration task, patients completed one or more runs of a BCI control task with each run consisting of 30 trials in which patients were instructed to attempt to use their EEG activity to open the exoskeleton by performing motor imagery and 30 trials in which patients were instructed to try to keep the exoskeleton in a closed position by resting. Each run of the BCI control task lasted about 10 minutes, patients completed 1-12 runs per day based upon their stamina and other time constraints. The 10-minute duration for each run of the BCI task was chosen as a realistic time period for patients to continuously focus. While patients varied quite a bit in the total time of use per day, because each day required the subjects to don the device and perform the calibration task prior to beginning BCI control and to remove and clean the system after a session, even completion of a single 10 minute run of the BCI control task required at least 40 minutes of total time to complete. Data from each calibration and control run was stored on the system and patients were instructed to maintain a log of their daily usage. Additionally, a wireless hotspot was used to upload anonymized EEG data to an online server. Data was analyzed by an experimenter and used to confirm that physiologic signals were recorded in order to provide feedback to patients about proper system usage.

Supplemental Data

Patient Characteristics

During the study, 23 patients were enrolled and 22 patients completed all 3 EEG screening sessions. 19 of the 22 patients demonstrated consistent movement-related EEG activity from the unaffected hemisphere ipsilateral to the affected hand. One patient demonstrated activations only contralateral to the affected hand, and two did not demonstrate consistent movement-related EEG activity. Of the 19 potential candidates that successfully completed the EEG screening, six patients did not continue with the study. This was due to the following reasons: 1) impaired cognitive understanding of the system that would have limited the ability to perform the necessary study procedures (1 patient), 2) the exoskeleton did not fit the patient's hand (1 patient), 3) conflicting personal commitments limiting regular usage (2 patients), and 4) health conditions that prohibited consistent use (2 patients). Therefore, 13 patients were eventually sent home with a BCI-driven exoskeleton system. During the study, three patients failed to comply with the study protocol by not utilizing the system at least five days per week and were discontinued from the study. Two of these patients were withdrawn due to an inability to meet the time commitments of the continued device usage and study visits. The third patient was withdrawn because of an unexpected move out of state. Because this study was designed to examine whether training with an powered exoskeleton driven through BCI control from the unaffected hemisphere could lead to functional improvements, data was only analyzed

from the 10 patients who complied with the study for the full 12-week period. While the study specifically focused on using the unaffected hemisphere to drive the BCI system, 8 of the 10 patients also demonstrated consistent movement-related spectral power changes in the ipsilesional hemisphere in addition to the contralesional hemisphere. Where possible the location and type of lesion were collected from patient medical records and are recorded in Table 1 of the main manuscript.

BCI System Usage

Supplemental Table I contains information describing the control features used by each patient, the number of runs of the BCI task performed by patients in their homes, and the characteristics of BCI control. Given the home-based context of non-expert electrode application and less controlled noisy environments when the system was being used during this study, careful attention was required when comparing BCI performance or EEG activity to metrics of motor recovery. Specifically, it was important to ensure that experimental runs without physiologic activity were excluded. Therefore, only BCI control runs with significant ($p<0.01$) r^2 values indicating differences in EEG activity between movement and rest were included for analysis of the relationship between ARAT changes and BCI performance and EEG activity. While over 50% of the BCI control runs were included in most patients, in a few patients (patients 3, 6, and 9), a larger percentage of BCI control runs were excluded.

Motor Function Changes

A detailed description of all outcome scores is shown in Supplemental Table II. At study onset, patients demonstrated moderate to severe motor impairments with ARAT scores ranging from 4-32. Similarly the patients had very low pinch strength scores, Motricity index scores, and while patients could generally perform flexion movements, they struggled to open their hand with no patient able to complete an extension movement to full extension. After the study there were significant ($p<0.05$) improvements in ARAT score, the grasp and grip subcomponents of ARAT score, Motricity index, grasp strength, and both the performance and satisfaction scores on the COPM.

As described in the manuscript, to establish the potential for BCI training to lead to functional improvements, a per-protocol analysis was used as the primary analysis. While it was not possible to collect completion data for the 3 patients that failed to complete the 12-week study period due to poor compliance, an intention-to-treat analysis was performed using the last ARAT score collected. Across the 13 patients sent home with a device, we observed a mean and median ARAT change of 5 and 5.5 points respectively which was highly significant ($p=0.002$).

Supplemental Table I. BCI control features used and characteristics of home-based BCI usage.

Patient	Affected UE	BCI Control Channel	BCI Control Frequency	Number of BCI Sessions (Days)	Number of BCI Runs	Number of BCI Runs Analyzed	Percent of BCI Runs Analyzed
1	R	C4	15 Hz	57	122	90	73.77%
2	L	C3	16 Hz	49	87	49	56.32%
3	R	C4	19 Hz	72	125	43	34.40%
4	R	C4	11 Hz	38	104	91	87.50%
5	R	C4	11 Hz	64	98	82	83.67%
6	L	C3	9 Hz	62	74	21	28.38%
7	L	C3	15 Hz	57	112	104	92.86%
8	L	C3	11 Hz	68	465	333	71.61%
9	R	C4	11 Hz	37	120	55	45.83%
10	L	C3	17 Hz	66	187	185	98.93%

Supplemental Table II. Summary of Outcome Measures.

Outcome Measure	Baseline Score	Exit Score	Score Change	p
**Grip Strength (lbs)**	14.70 (15.80) ± 6.86	18.03 (18.30) ± 7.67	3.32 (2.70) ± 4.23	***0.046***
**Pinch Strength (lbs)**	1.68 (0.00) ± 2.23	4.38 (0.50) ± 5.40	2.70 (0.50) ± 4.73	0.125
**Motricity Index**	39.8 (37.0) ± 15.5	51.9 (51.0) ± 19.9	12.1 (12.0) ± 13.4	***0.027***
Motricity Index (Pinch)	7.4 (5.5) ± 8.5	13.9 (11.0) ± 13.6	6.5 (0) ± 10.12	0.125
Motricity Index (Elbow)	17.9 (19.0) ± 4.8	21.2 (25.0) ± 4.8	3.3 (5.5) ± 5.5	0.13
Motricity Index (Shoulder)	13.4 (14.0) ± 3.9	15.8 (14.0) ± 3.8	2.3 (0.0) ± 2.8	0.125
**ARAT Total**	13.4 (10.1) ± 10.25	19.6 (16.0) ± 12.2	6.2 (6.0) ± 4.4	***0.002***
ARAT Grasp (Max=18)	4.3 (2.75) ± 4.3	6.7 (6.0) ± 4.7	2.4 (2.0) ± 2.1	***0.016***
ARAT Grip (Max=12)	3.6 (3.0) ± 2.3	5.4 (4.5) ± 3.0	1.9 (1.5) ± 1.6	***0.004***
ARAT Pinch (Max=18)	1.5 (0.0) ± 2.8	2.3 (0.0) ± 4.2	0.9 (0.0) ± 1.5	0.250
ARAT Gross (Max=9)	4.1 (4.0) ± 1.7	5.2 (5.5) ± 1.7	1.1 (0.0) ± 1.4	0.125
**Modified Ashworth Scale**	1.17 (1.25) ± 0.76	1.28 (1.00) ± 0.97	0.11 (0.00) ± 0.75	0.875
**Active Range of Motion**				
Flexion (Digits 2 & 3)	66.7 (70.75) ± 11.8	72.9 (75.0) ± 20.1	6.3 (6.25) ± 10.1	0.099
Extension (Digits 2 & 3)	49.1 (36.75) ± 21.0	47.1 (55.0) ± 31.2	-2.0 (-9.25) ± 25.4	0.819
Flexion (Digits 4 & 5)	63.6 (67.5) ± 19.2	69.4 (75.0) ± 22.4	5.8 (8.75) ± 11.8	0.179
Extension (Digits 4 & 5)	41.8 (44.75) ± 22.6	42.8 (40.0) ± 29.9	1.1 (-1.0) ± 21.0	0.884
**Canadian Occupational Performance Measure (COPM)**				
Performance	2.10 (2.0) ± 1.08	3.66 (3.3) ± 1.68	1.56 (1.6) ± 1.70	***0.022***
Satisfaction	1.26 (1.1) ± 0.43	2.80 (2.0) ± 2.10	1.54 (0.8) ± 1.86	***0.031***

a. All measures are reported as mean (median) ± SD

Supplemental References

1. Marple SL. *Digital spectral analysis : With applications*. Englewood Cliffs, N.J.: Prentice-Hall; 1987.
2. Lyle RC. A performance test for assessment of upper limb function in physical rehabilitation treatment and research. *Int J Rehabil Res*. 1981;4:483-492
3. Yozbatiran N, Der-Yeghiaian L, Cramer SC. A standardized approach to performing the action research arm test. *Neurorehabil Neural Repair*. 2008;22:78-90
4. Lang CE, Bland MD, Bailey RR, Schaefer SY, Birkenmeier RL. Assessment of upper extremity impairment, function, and activity after stroke: Foundations for clinical decision making. *J Hand Ther*. 2013;26:104-114;quiz 115
5. Lang CE, Wagner JM, Dromerick AW, Edwards DF. Measurement of upper-extremity function early after stroke: Properties of the action research arm test. *Arch Phys Med Rehabil*. 2006;87:1605-1610
6. Rabadi MH, Rabadi FM. Comparison of the action research arm test and the fugl-meyer assessment as measures of upper-extremity motor weakness after stroke. *Arch Phys Med Rehabil*. 2006;87:962-966
7. Bodiam C. The use of the canadian occupational performance measure for the assessment of outcome on a neurorehabilitation unit. *The British Journal of Occupational Therapy*. 1999;62:123-126
8. Collin C, Wade D. Assessing motor impairment after stroke: A pilot reliability study. *J Neurol Neurosurg Psychiatry*. 1990;53:576-579
9. Pandyan AD, Johnson GR, Price CI, Curless RH, Barnes MP, Rodgers H. A review of the properties and limitations of the ashworth and modified ashworth scales as measures of spasticity. *Clin Rehabil*. 1999;13:373-383
